# Natural fish oil improves the differentiation and maturation of oligodendrocyte precursor cells to oligodendrocytes *in vitro* after interaction with the blood–brain barrier

**DOI:** 10.3389/fimmu.2022.932383

**Published:** 2022-07-22

**Authors:** Paweł Piatek, Natalia Lewkowicz, Sylwia Michlewska, Marek Wieczorek, Radosław Bonikowski, Karol Parchem, Przemysław Lewkowicz, Magdalena Namiecinska

**Affiliations:** ^1^ Department of Immunogenetics, Medical University of Lodz, Lodz, Poland; ^2^ Department of Periodontology and Oral Mucosal Diseases, Medical University of Lodz, Lodz, Poland; ^3^ Laboratory of Microscopic Imaging and Specialized Biological Techniques, Faculty of Biology and Environmental Protection, University of Lodz, Lodz, Poland; ^4^ Department of Neurobiology, Faculty of Biology and Environmental Protection, University of Lodz, Lodz, Poland; ^5^ Faculty of Biotechnology and Food Sciences, Institute of Natural Products and Cosmetics, Lodz University of Technology, Lodz, Poland; ^6^ Department of Food Chemistry, Technology and Biotechnology, Faculty of Chemistry, Gdansk University of Technology, Gdansk, Poland

**Keywords:** long-chain fatty acids, Blood-brain barrier, astrocytes, endothelial cells, oligodendrocyte precursor cells, oligodendrocytes, remyelinating therapy

## Abstract

The blood–brain barrier (BBB) tightly controls the microenvironment of the central nervous system (CNS) to allow neurons to function properly. Additionally, emerging studies point to the beneficial effect of natural oils affecting a wide variety of physiological and pathological processes in the human body. In this study, using an *in vitro* model of the BBB, we tested the influence of natural fish oil mixture (FOM) *vs*. borage oil (BO), both rich in long-chain polyunsaturated fatty acids (LC-PUFAs) and monounsaturated fatty acids (MUFAs) such as oleic acid (C18:1n9c) or nervonic acid (NA), on human oligodendrocyte precursor cells (hOPCs) during their maturation to oligodendrocytes (OLs) regarding their ability to synthesize myelin peptides and NA. We demonstrated that FOM, opposite to BO, supplemented endothelial cells (ECs) and astrocytes forming the BBB, affecting the function of hOPCs during their maturation. This resulted in improved synthesis of myelin basic protein (MBP), myelin oligodendrocyte glycoprotein (MOG), proteolipid protein (PLP), and NA in mature OLs. This effect is probably the result of BBB cell and hOPC stimulation *via* free fatty acid receptors (FFARs), which increases insulin growth factor-1 (IGF-1), ciliary neurotrophic factor (CNTF), and brain-derived neurotrophic factor (BDNF) and inhibits fibroblast growth factor 2 (FGF-2) synthesis. The unique formula of fish oil, characterized by much more varied components compared to those of BOs, also improved the enhancement of the tight junction by increasing the expression of claudin-5 and VE-cadherin on ECs. The obtained data justify consideration of naturally derived fish oil intake in human diet as affecting during remyelination.

## Introduction

The functional and structural integrity of the blood–brain barrier (BBB) as the pivotal feature to maintain the homeostasis of the brain microenvironment has recently been widely considered. Among others, maintaining the BBB integrity and proper myelin forming by oligodendrocytes (OLs) are crucial in proper central nervous system (CNS) functioning. A deterioration in BBB function may play a significant role in the pathogenesis of disease, as the BBB dynamically responds to many events associated with flow disturbances. Physiologically, BBB permeability is strictly regulated by cell–cell interactions and cell-derived bioactive factors (1). Its static function depends on endothelial tight junctions (TJs) and the basal lamina. In turn, the TJ is formed by TJ-related proteins including claudin-5 (CLDN-5), occludin (OCLN), zonula occludens (ZO), and VE-cadherin (VEC) (2, 3), which are important markers of BBB tightness. On the other hand, axonal loss and functional disability in forming myelin sheaths by OLs within the CNS are important processes responsible for the growing list of neurological diseases from autoimmunological diseases such as multiple sclerosis, viral infection, and metabolic (deficiency of minerals or vitamins), toxic, traumatic, and neoplastic disorders to rare genetic disorders such as Canavan’s or Alexander’s disease (4, 5). Therefore, the restoration of the neuron within the CNS and in the peripheral nervous system is the major therapeutic goal in human myelin disorders. Although the direct cause of myelin pathological morphology is different within the course of these diseases, many studies point out that limited remyelination disturbing the natural process of CNS regeneration can be compensated by supplying the necessary substrates for myelin synthesis (6, 7). The proper OL production of myelin requires not only appropriate substrates for its synthesis but also an extracellular environment formed, among others, by BBB cells, glia cells, remyelinating neurons, and external signals (8). The myelin structure, characterized by a high lipid-to-protein ratio, contains at least 70% lipids on a dry matter basis, including cholesterol (27.7%), galactolipid galactosylceramide (GalC) (22.7%), and sulfatide (3.8%) (9). GalC and sulfatide are highly ordered with long saturated and monosaturated fatty acyl chains containing 22–26 carbon atoms (10). Among the proteins, the most essential are proteolipid protein (PLP), myelin basic protein (MBP), and myelin oligodendrocyte glycoprotein (MOG). The interactions between both lipids and proteins are fundamental for myelin formation, regulating protein transport and the molecular organization within the myelin sheath (11). Thus, lipids control protein sorting, while myelin proteins are able to arrange lipids, creating regions of specialized molecular packing (e.g., lipid rafts) related to their functioning. The observed alterations in protein–lipid trafficking, misfolding in conformational changes of myelin proteins, or changes within the timing of the myelination machinery may cause severe pathoneurological consequences. Therefore, pharmacological intervention should be compatible with myelin synthesis of all components and not disturb the natural mechanisms of oligodendrocyte precursor cell (OPC) polarization to mature OLs.

In this context, many studies focused on the benefits of natural oils, rich in essential monounsaturated long-chain fatty acids (FAs), mainly nervonic acid [NA; C24:1(n9)], as necessary components of sphingolipids, representing approximately 35% of total myelin lipids. NA is found in the seed oils of some wild plants, including *Lunaria annua* (honesty), *Acer truncatum* (purpleblow maple), *Tropaeolum speciosum* (flame flower), *Borago officinalis* (borage), and *Cannabis sativa* (hemp) (3), and in fish oils (12). The pathway of NA biosynthesis is a complicated process, which includes the elongation step from palmitic acid (C16:0) and stearic acid (C18:0) by desaturation to monounsaturated fatty acids (MUFAs) as palmitoleic acid [C16:1(n-9)] and oleic acid [C18:1(n-9)]. Although NA biosynthesis proceeds readily in the brain, some studies indicated the use of NA supplements and substrates for its synthesis has become valuable and desirable in the treatment of several neurological disorders (13). Many clinical studies are also directed on polyunsaturated fatty acids (PUFAs) n-3 especially eicosapentaenoic acid [EPA 20:5(n-3)] and docosahexaenoic acid [DHA 22:6(n-3)] and their beneficial effects associated with their neuroprotective and anti-inflammatory properties in a variety of neurodegenerative and neurological diseases (14, 15). It has been shown that DHA promotes progenitor cell differentiation into neural cells, and its adequate level in neural cell membranes is essential for their proper functioning (16, 17). Additionally, EPA and DHA were demonstrated to cross the BBB by simple diffusion, mediating the neuroprotection process through prolonging the life span of glia cells and inhibiting microglia and inflammatory cells (18).

In our previous studies, we demonstrated that natural fish oil, rich in MUFAs such NA and oleic acid (C18:1n9c), PUFAs (EPA and DHA), and saturated FAs [palmitic acid (C16:0)], improves the ability of mature OLs to synthesize myelin peptides (MBP, PLP, MOG) and direct lipid metabolism on the NA pathway, simultaneously inhibiting arachidonic acid [C20:4(n-6)]-dependent products (7). These data indicate that a natural lipid mixture containing EPA/DHA, palmitic acid, and NA not only may affect the immune cells to restrict inflammation but also actively participates in myelin synthesis, acting comprehensively in CNS regeneration. The CNS is classically considered as being autonomous in lipid metabolism. Therefore, it is an open question whether FAs derived from food and present in the serum will also have a positive effect on OPCs being in direct contact with BBB-forming cells. Under physiological conditions, the BBB limits the influx of intravascular contents including lipoprotein particles that do not cross the BBB, so the lipids needed for myelin synthesis must be produced *de novo* in the CNS. In light of recent advances, CNS myelin membrane synthesis not only requires endogenous OL lipid synthesis, but also depends on extracellular lipids provided by astrocytes. Furthermore, when astroglia lipid synthesis is selectively compromised, OLs incorporate circulating lipids into the myelin membrane. Interestingly, a lipid-rich diet rescues hypomyelination by circulating lipid flowing into the myelin membrane under conditions of disturbed astrocyte metabolism (19).

As the CNS is considered to be autonomous in lipid metabolism being protected from lipids in the circulation by the BBB, we consider two probable mechanisms of PUFA and MUFA action: indirect by activating astrocytes and endothelial cells (ECs) forming the BBB to release some growth factors and direct influence on maturating OLs. There is no research on whether natural oils used in the diet affect the BBB tightness and maturating OPCs and the ability of mature OLs to synthesize myelin proteins and sphingolipids. Comparing results from two natural oils of different origins (fish and plants), we aimed to justify the need to take the oil with better effect on the remyelination process.

## Materials and methods

### Study design

The BBB model of maturating human oligodendrocyte precursor cells (hOPCs) *in vitro* was used to estimate the properties of the natural oils, registered as a medical food, in CNS renewal. Two naturally derived oils, which are characterized by similar percentages of palmitic acid (C16:0), oleic acid [C18:1(n-9c)], and NA [C24:1(n9)] as the main components involved in sphingomyelin synthesis, representing different kingdoms of life (animal and plant) (**Supplementary Table S1**) were used. The natural fish oil mixture (FOM) and borage oil (BO) were added directly to separated BBB cells during OPC maturation in the amount of 5% v/v.

### Blood–brain barrier *in vitro* model

We used the BBB *in vitro* model from Niego and Medcalf (20) with our own modifications. Tissue-culture inserts were placed (1.13-cm^2^ area with polyester porous membrane, 3-µm pore size) in the wells of the 6-well plate. The luminal surface of the inserts was coated overnight in a humidified 37°C incubator with rat collagen I [20 µg/cm^2^; 50 µl of 132 µg/ml collagen I in 0.02% acetic acid in Milli-Q water (MQH_2_O)]. Then, inserts were washed (both from the abluminal and luminal side) with MQH_2_O to remove the residual acid. In the next step, the inserts were inverted and silicone tubes were gently fitted around the rim of the porous membrane to create new essential well above the abluminal surface. The underside of the insert needs to be sealed to avoid medium leakage. Next, the silicone plugs were placed into the luminal cavity. Then, 15 × 10^4^ SVG p12 cells were seeded in 700-µl SVG p12 medium directly into the silicone well above the abluminal membrane. In order to monitor the adherence state of the cells, the same astrocyte suspension was seeded into a standard 24-well plate. When astrocytes were sufficiently adhered in the control 24-well plate, inserts were transferred to the normal orientation. Then, 7.5 × 10^4^ hCMEC/D3 cells were seeded in 700 μl on the collagen-coated luminal surface. SVG and p12/hCMEC coculture was incubated for 3 days in the medium without medium exchange before the next set of experiment (5% CO_2_, 37°C, 95% humidity).

At the bottom of the 12-well plate, 1 × 10^6^ MO3.13 cells were seeded and incubated for 24 h with 5% CO_2_, 37°C, 95% humidity. In the next step, the previously prepared BBB *in vitro* model was used. Inserts were put into each of the 12 wells with OPCs, and 5% v/v oils with natural NA were added to some parts of the inserts and 100 nM phorbol 12-myristate 13-acetate (PMA) to chosen wells and then incubated for 7 days with medium changing after 72 h.

### Human cell model of progenitor cell differentiation to mature myelin-producing oligodendrocytes

A human oligodendroglia cell line MO3.13 (OLs, Tebu-bio, Le Perray En Yvelines, France) was used as the model of progenitor cell differentiation to mature myelin-producing OLs. We chose the MO3.13 cell line, which differentiates after PMA stimulation, as the most adequate model of OPC maturation. Unlike other OL lines (human oligodendroglioma or KG-1C), MO3.13 cells during maturation exhibited the strongest similarity to primary human OLs in morphology and in gene and protein expression (21). OLs were cultured in DMEM–high-glucose medium supplemented with 10% fetal bovine serum (FBS) and 1% penicillin-streptomycin and maintained at 37°C with 5% CO_2_ in a humidified atmosphere. The cultures were conducted in 75-cm^2^ flasks (Nunc, Thermo Scientific). After 80% confluence, cells were passaged using a 0.25% trypsin–EDTA solution in total three times for a week with a dilution factor of 1/8. To induce OPC maturation, PMA was added (100 mM) for 72 h, and cells were incubated at 37°C with 5% CO_2_ in humidified atmosphere. All of the reagents used for culture were purchased from Sigma-Aldrich. The purity and cell differentiation were estimated by OL marker O4, nestin, MOG, and glial fibrillary acidic protein (GFAP) expression by immunocytochemical (ICC) analysis, as well as morphological shape in differential contrast microscopy (DIC) examination (**Supplementary Figure S1B**).

### Exposition of naturally derived oils on maturating hOPCs *via* blood–brain barrier cells

FOM derived from *Centroscymnus crepitater*, *Etmopterus granulosus*, *Deania colceai*, *Centrophorus scalpratus*, *Sardinops sagax*, *Scomber scombrus*, and *Gadus morhua* species and BO (*Borago officinalis*) were used to examine the effect of fish-derived oils and plant-derived oils on OPC differentiation and myelin synthesis. FOM contained the following: 9.2% of palmitic acid (C16:0), 0.4% of stearic acid (C18:0), 19.8% of oleic acid (C18:1n9c), 6.1% of cis-Eicos-11-enoic acid (C20:1n9), 1.1% of erucic acid (C22:1n9), and 0.9% of NA [C24:1(n-9)]. (UPRP patent # P.416768) BO contained the following: 9.8% of palmitic acid (C16:0), 4.3% of stearic acid (C18:0), 19.0% of oleic acid (C18:1n9c), 3.2% of cis-Eicos-11-enoic acid (C20:1n9), 1.2% of erucic acid (C22:1n9), and 0.95% of NA [C24:1(n-9)]. The complete oil composition determined by gas chromatography/mass spectrometry (GC/MS) is summarized in **Supplementary Table S1**. At 24 h before the experiments, 2 × 10^6^ MO3.13 cells were seeded on six-well plates in DMEM–high-glucose medium supplemented with 10% FBS and 1% penicillin-streptomycin at 37°C with 5% CO_2_. The next day, medium was changed (5% FBS, 1% penicillin-streptomycin) and PMA (100 nM) was used as a stimulator of OPC differentiation. In parallel experiments, 5% FOM or 5% BO was added to the culture for 24–72 h. After incubation, supernatants were collected, and cells were washed in phosphate-buffered saline (PBS) for further analysis.

### Immunocytochemical analysis

For ICC analysis, cells were transferred to gelatin-coated microscope slides by cytospin (300 × g, 10 min) and fixed with 4% formaldehyde solution for 20 min at 21°C. Fixed cells were washed with PBS and blocked with 10% rabbit blocking serum (Santa Cruz Biotechnology, Dallas, TX, USA) supplemented with 3% TritonTM X-100 (Sigma-Aldrich, St. Louis, MO, USA) for 45 min at 21°C. Next, they were washed and double-stained for MOG/MBP, PLP, CLDN-5/VEC, free fatty acid receptor FFAR4/FFAR2, FFAR1/FFAR3, GFAP/phalloidin, O4/phalloidin, and nestin/PLP. Anti-CLDN-5 (1:100, Biorbyt, Cambridge, UK), anti-VEC (BV6, 5 μg/ml, Enzo Life Sciences), anti-FFAR1 (GPR40, 10 μg/ml, Cayman Chemical), anti-FFAR2 (GPR43, 1:100, Cayman Chemical), anti- FFAR3 (GPR41, 1:50, Proteintech, Manchester, UK), anti-FFAR4 (GPR120, clone H-10, 1:50, Santa Cruz Biotechnology, Dallas, TX, USA), anti-MOG (clone D-2, 1:100, Santa Cruz Biotechnology), anti-MBP (clone F-8, 1:300, Santa Cruz Biotechnology), anti-PLP (clone G-17, 1:100, Santa Cruz Biotechnology), anti-O4 (1 μg/ml, clone # O4, R&D Systems), anti-nestin (clone 10C2, 2 μg/ml, BioLegend), phalloidin (F-actin)/Texas Red (1 μg/ml, Invitrogen), anti-GFAP (clone H-50, 1:100, Santa Cruz Biotechnology), and rat IgG2b kappa (eB149/10H5, eBioscience) as negative isotype control were used. All antibodies were suspended in PBS supplemented with 1.5% blocking rabbit serum, 0.3% Triton X-100, and 0.01% sodium azide and incubated overnight at 4°C. Cells were washed, and secondary fluorescent Abs were added for 1 h at room temperature: goat pAb to mouse TR (5 μg/ml, cat. T862, Invitrogen, USA) with goat pAbs to rabbit FITC (2 μg/ml, Invitrogen, USA) or goat pAb to mouse FITC (1:100, Abcam) with goat pAbs to rabbit TR (4 μg/ml, Invitrogen, USA). For nuclei DNA staining, DAPI (4′,6-diamidino-2-phenylindole, 1.5 μg/ml, UltraCruz Mounting Medium, Santa Cruz Biotechnology, Dallas, TX, USA) was used. The confocal laser scanning microscopy platform TCS SP8 (Leica Microsystems, Germany) with the objective ×63/1.40 (HC PL APO CS2, Leica Microsystems, Germany) was used for microscopic imaging. Leica Application Suite X (LAS X, Leica Microsystems, Germany) was used for cell imaging. Fluorescence intensity was determined in the Region of Interest as the sum of the fluorescence from all segments (bordered by the line) divided by their number [arbitrary units (a.u.)]. The average fluorescence was calculated using at least 100 single cells for each sample. Nonspecific fluorescence (signal noise) was electronically diminished to level when nonspecific signal was undetectable (background).

### Differential interference contrast microscopy

The visualization of live cell morphology was performed on 8-well glass chamber slides (Nalge Nunc International, Waltham, MA, USA). During the course of MO3.13 polarization to mature OLs within 72 h, cells were imaged with a Zeiss Axiovert 200 inverse microscope with a Zeiss Plan Neofluar ×40/0.6/Ph2 Korr differential interference contrast objective (Göttingen, Germany).

### ELISA method

Human BDNF, CNTF, FGF-2, and IGF-1 (all from Wuhan EIAab Science, Wuhan, China) were measured in supernatants using ELISA kits. The detection limits were as follows: 0.33 pg/ml (BDNF and CNTF), 12 pg/ml (IGF-1), and 1.56 pg/ml (basic FGF-2). All samples were analyzed at the same time in duplicate.

### RNA isolation

Total RNA was extracted from human OLs with a mirVanaTM miRNA Kit (Thermo Fisher Scientific). After isolation, the RNA concentration and the purity analyses were performed by NanoDrop 2000 (Thermo Scientific).

### mRNA analysis of myelin basic protein, myelin oligodendrocyte glycoprotein, and proteolipid protein expressed in oligodendrocytes by droplet digital PCR

RNA was transcribed into cDNA using iScript Reverse Transcription Supermix (Bio-Rad, USA). cDNA quantification of MBP, MOG, and PLP in hOPCs was done with specific TaqMan gene expression probes (UniGene: Hs00921945_m1, Hs01555268_m1, and Hs00166914_m1, respectively) using EvaGreen Digital PCR Supermix with Droplet Generation Oil for EvaGreen and the QX200 droplet digital PCR system (ddPCR; Bio-Rad Quanta SOFT Analysis Pro v.1.0.596 software, Hercules, CA, USA). The results were expressed as the number of gene copies per 20 symbol microliter of mixture reaction.

### Lipid profiling for free and esterified fatty acids by gas chromatography/mass spectrometry analysis

To the 50 mg of lyophilized samples, 200 µl of methyl tert-butyl ether (Sigma-Aldrich by Merck, Darmstadt, Germany) and 200 µl of a 0.25-M solution of trimethylsulfonium hydroxide in methanol (Sigma-Aldrich) were added. After that, the samples were incubated at 80°C for 30 min, and GC/MS was performed by GC/MS Pegasus 4D (LECO Corp., St. Joseph, MI, USA). The components were directly injected into the port of a GC/MS. The GC column Rt-2560 (100 m × 0.25 mm, 0.20 µm, Restek Corp., Bellefonte, USA, cat. No. 40602) was used. Then, 1 µl of the sample was applied to the split/splitless (SSL) injector in splitless mode (injector temperature 240°C). The GC oven temperature was initially held at 140°C for 5 min and then increased to 240°C for 30 min at a rate of 4°C/min. Helium as a carrier gas was used at a flow of 1 ml/min. Mass spectra were collected using a time-of-flight mass spectrometer. The settings of mass spectrometry were as follows: ion source temperature 200°C, ionization energy 70 eV, and scan range 33–550 atomic mass unit (amu). The obtained mass spectra were compared with those of NIST/EPA/NIH and Wiley Registry of Mass Spectral Data mass spectral libraries.

### Reverse-Phase Liquid Chromatography Q-Orbitrap High-Resolution Mass Spectrometry analysis of lipid species in fish oil mixture and borage oil

The FOM and BO were dissolved in chloroform (10 mg/ml) followed by dilution in acetonitrile (1:9 v/v). The lipid species were chromatographic separated using a Dionex Ultimate 3000 UHPLC system (Thermo Scientific™, Dionex, San Jose, CA, USA) equipped with a Kinetex XB-C18 column (50 mm × 2.1 mm, 1.7 μm, Phenomenex, Torrance, CA, USA). The mobile phases consisted of acetonitrile/water (50:50 v/v) with 10 mM ammonium formate and 0.1% formic acid (phase A) and 2-propanol/acetonitrile/water (85:10:5 v/v/v) with 10 mM ammonium formate and 0.1% formic acid (phase B). All reagents, 2-propanol, acetonitrile, ammonium formate, formic acid (all LC-MS grade), and chloroform (HPLC gradient grade) were purchased from Merck Darmstadt, Germany. High-purity water was produced using Millipore Elix 5 purification system (Merck, Darmstadt, Germany). The gradient program was set as follows: linear increase from 32% to 70% B at 11 min followed by linear increase to 97% B at 18 min and then isocratically held for 12 min (30 min). Finally, the initial mobile phase composition was set, and the column was equilibrated for 6 min. The flow rate was 260 μl/min, and the column temperature was 50°C. The injection volume was 5 μl.

The chromatographic system was coupled to the Q Exactive™ Focus Hybrid Quadrupole-Orbitrap mass spectrometer (Thermo Fisher Scientific, Bremen, Germany) equipped with a heated electrospray ionization source (HESI II). The ionization parameters in positive ion mode were as follows: spray voltage 3.5 kV, capillary temperature 230°C, heater temperature 300°C, sheath gas flow rate 45 a.u., auxiliary gas flow rate 15 a.u., and S-lens RF level 35. The full-scan analysis parameters were as follows: resolution 70,000, AGC target 1e6, max injection time 100 ms, and scan range 200–2,000 *m/z*. The data-dependent MS^2^ parameters were as follows: resolution 17,500, isolation window 3.0 *m/z*, and normalized collision energy 30. The mass spectrometer was tuned and calibrated using a calibration solution (Thermo Scientific) containing n-butylamine, caffeine, Met-Arg-Phe-Ala (MRFA), and Ultramark 1621. The untargeted analyses of lipid species present in the FOM and BO samples were carried out using MS-DIAL software (22).

### Statistics

Arithmetic means and standard deviations were calculated for all parameters. A statistical analysis of differences was performed using the one-way ANOVA test. Scheffe’s test was used for multiple comparisons as a *post-hoc* test when statistical significances were identified in the ANOVA test. The differences in the adequate groups between “direct exposition” and “BBB model” were calculated using the paired t-test. *p* ≤ 0.05 was considered as a significant difference.

## Results

### Blood–brain barrier cells supplemented by fish oil mixture enhanced myelin oligodendrocyte glycoprotein, proteolipid protein, and myelin basic protein synthesis by mature oligodendrocytes

**Figure 1:**
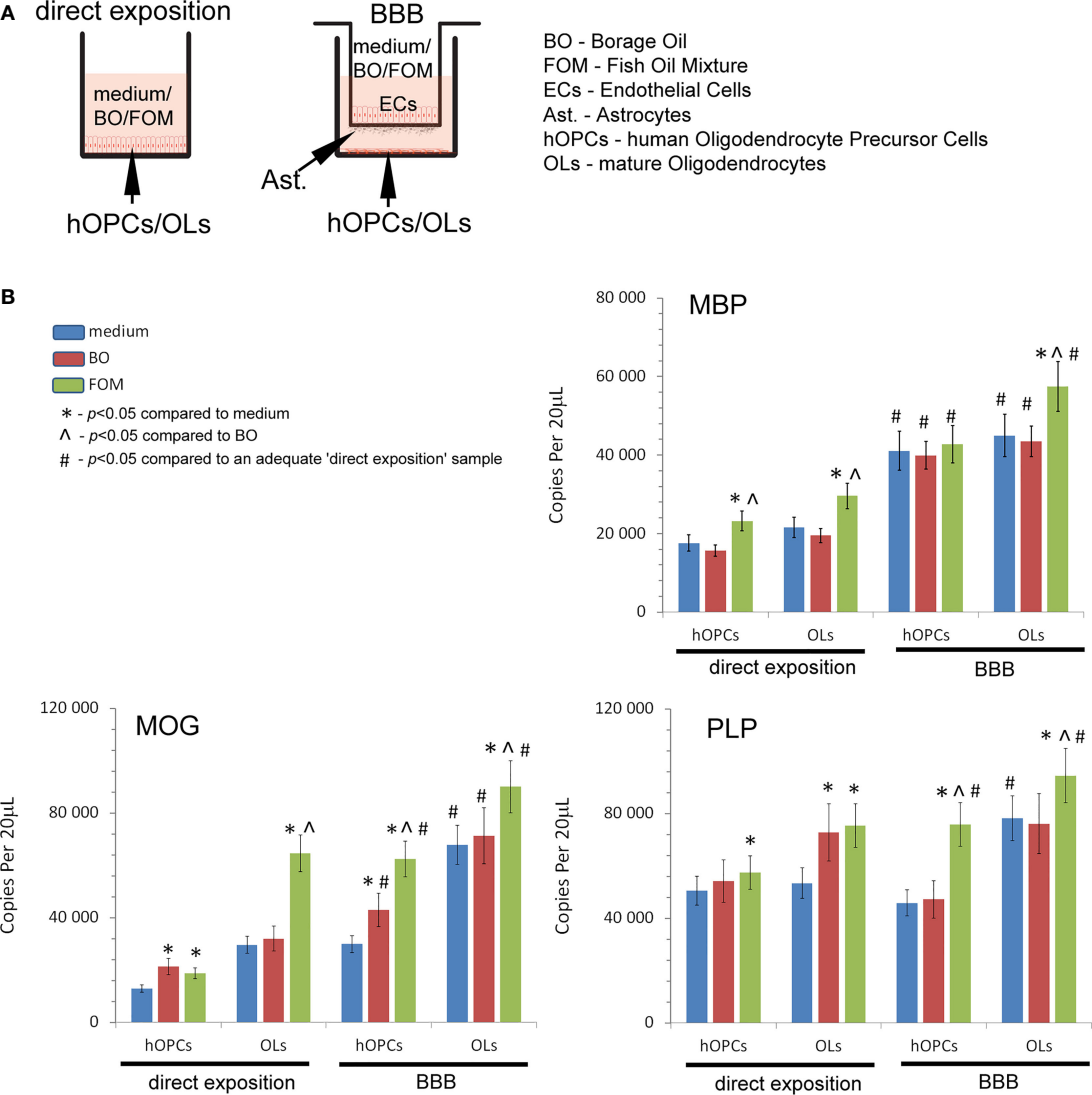
Quantitative analysis of the mRNA expression of myelin peptides revealed that hOPCs supplemented with fish oil mixture (FOM) contrary to borage oil (BO) during maturation results in enhanced transcription of MOG, PLP, and MBP in mature OLs. This synthesis was further improved by the use of the blood–brain barrier (BBB). **(A)** Diagram of the experiment performed to characterize the role of the BBB exposed to FOM or BO and their effect on OPC differentiation into mature MBP/PLP/MOG-producing OLs. **(B)** The quantitative mRNA analysis of MBP, MOG, and PLP expressed by hOPCs and OLs. The results were expressed as the number of gene copies per 20 μl of samples. The graph presented the means ± SD from three independent experiments. The differences between medium/BO/FOM were calculated using the one-way ANOVA test and Scheffe’s test. The differences in the adequate groups between “direct exposition” and “BBB model” were calculated using the paired t-test.

In the first set of experiments, we examined the influence of fish oil mixture (FOM) and borage oil (BO) on myelin protein synthesis by mature OLs. Using the *in vitro* model of hOPC maturation, we investigated the effect of FOM and BO on myelin peptide synthesis by direct exposition and with the BBB model to mimic the natural process of hOPCs during their maturation to OLs (**Figure 1A**). Quantitative analysis of mRNA gene copies of myelin proteins revealed that MBP, MOG, and PLP synthesis by OLs was significantly increased with the use of BBB cells. We noted 22,590 copies/20 μl without BBB *vs*. 44,940 copies/20 μl with BBB for MBP, 24,590 *vs*. 67,900 for MOG, and 53,430 *vs*. 78,200 for PLP. The engagement of BBB in the stimulation of MBP, MOG, and PLP by mature OLs was additionally enhanced by FOM supplementation, contrary to that of BO, by over 20%. We noted the average increase in MBP to 57,410 copies/20 μl (27%), in MOG to 90,107 copies/20 μl (32%), and in PLP to 94,500 copies/20 μl (21%). The effect on all myelin protein synthesis enhancements was also observed in direct exposition of OLs or OPCs to FOM that confirms our previous research (7). In direct and in the BBB model of exposition to BO, we observed the increase only in MOG mRNA copies in hOPCs. In direct exposition, we noted 12,820 (medium) *vs*. 21,320 copies/20 ml (BO supplementation), while in the BBB model, 29,880 (medium) *vs*. 62,900 copies/20 ml (BO supplementation) (**Figure 1B**).

**Figure 2:**
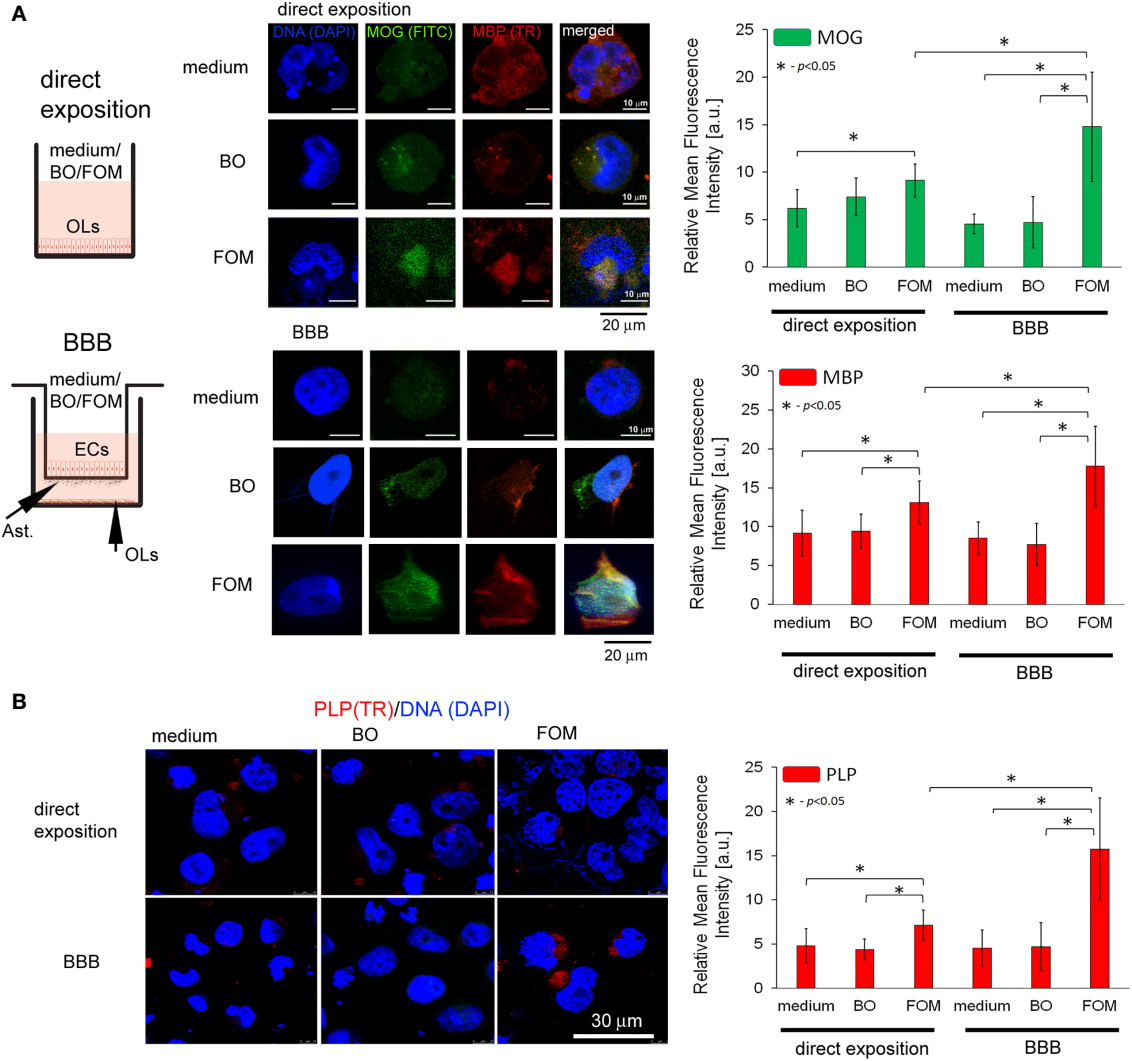
The supplementation of BBB cells by FOM, opposite to BO, during hOPC maturation enhanced MOG, PLP, and MBP synthesis by mature OLs. The use of the BBB increased the ability of mature OLs to synthesize myelin peptides compared to direct exposition of hOPCs to FOM during their maturation. (**A**, left panel) Diagram of the experiment performed to characterize the role of the BBB exposed to FOM or BO and their effect on OPC differentiation into mature MBP/PLP/MOG-producing OLs. (**A**, right panel) The ICC analysis of MOG (green pseudocolor) and MBP (red). **(B)** PLP immunocytochemical analysis. The data from the quantitative fluorescence signal of protein analysis are presented as the relative mean fluorescence intensity [arbitral units (a.u.)] ± SD from three independent experiments using at least 100 single cells for each test. The differences between medium/BO/FOM were calculated using the one-way ANOVA test and Scheffe’s test. The differences in the adequate groups between “direct exposition” and “BBB model” were calculated using the paired t-test.

Subsequently, we analyzed the effect of FOM and BO supplementation of BBB and direct supplementation of maturating hOPC on the intracellular level of MOG, PLP, and MBP peptides in OLs using the ICC method. First, we noted that direct exposition of hOPC on FOM during maturation results in increased MBP, MOG, and PLP expression in mature OLs compared to non-treated cells (MBP by about 23%, MOG 20%, and PLP 48%). FOM used in the BBB system resulted in an additional significant increase of MBP and MOG synthesis (**Figure 2A**) as well as PLP (**Figure 2B**) in comparison to its direct exposition. We noted the average increase in MBP to be about 41%, MOG 86%, and PLP 121% compared to direct exposition. We did not observe any changes in MBP, MOG, and PLP synthesis after BO supplementation with direct exposition and in the BBB model (**Figures 2A, B**). Astonishingly, in the set of experiments without oil exposition, we did not observe the influence of BBB cells on the intracellular expression of the tested myelin peptides in 72-h incubation as it was observed in mRNA expression, suggesting that BBB cells preactivate OLs to more efficient myelin production but do not trigger their synthesis.

To summarize, this set of experiments revealed that BBB cells are necessary for preparing hOPCs to more intensive myelin peptide synthesis by mature OLs. BBB conditioned by FOM, contrary to BO, during maturation of hOPCs into mature MBP/PLP/MOG-producing OLs improved their ability to synthesize these peptides.

### Blood–brain barrier supplementation by fish oil mixture during polarization of human oligodendrocyte precursor cells to mature oligodendrocytes results in increased insulin growth factor-1, brain-derived neurotrophic factor, and ciliary neurotrophic factor but decreased fibroblast growth factor 2 concentration

The juxtaposition of the experiments presented a significant role of BBB in myelin peptide synthesis improvement by OLs without direct contact of astrocytes with OLs, suggesting the growth factor engagement. As the differentiation of OPCs into mature myelinating OLs is a multistep process, tightly controlled by specific environmental factors, from their wide spectrum, we have chosen these ones released by astrocytes and ECs of the BBB and maturating OPCs, which can influence on OLs. In the literature, brain-derived neurotrophic factor (BDNF), insulin growth factor-1 (IGF-1), ciliary neurotrophic factor (CNTF), and fibroblast growth factor 2 (FGF-2) are proven to be particularly important in the maturation of hOPCs and neuronal regeneration (23, 24).

**Figure 3:**
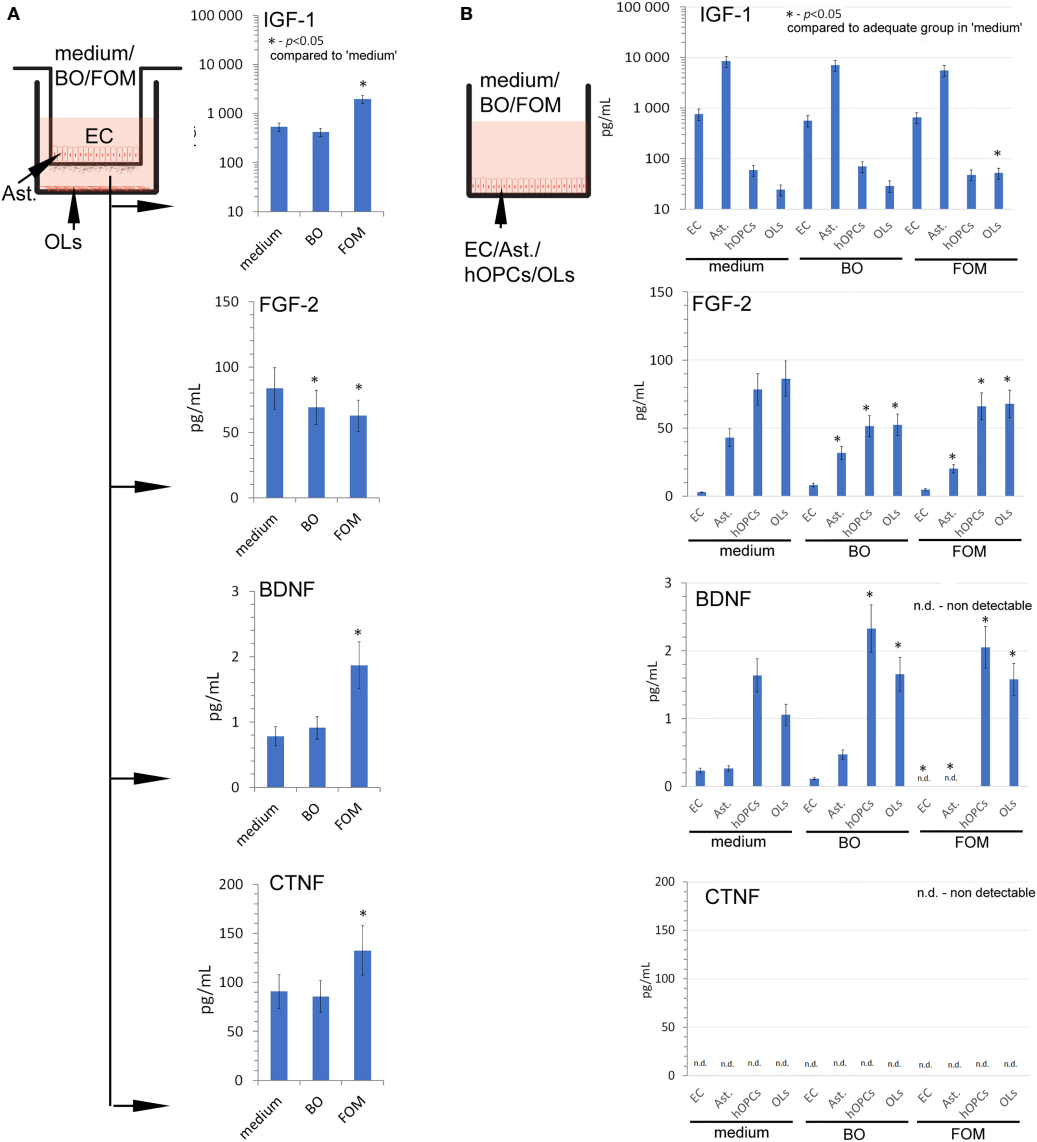
The supplementation of BBB cells by FOM during polarization of hOPCs to mature OLs resulted in the stimulation of IGF-1, BDNF, and CNTF but decreased FGF-2 synthesis. **(A)** The concentration of IGF-1, BDNF, CNTF, and FGF-2 in supernatants of the BBB construct with OLs. BBB cells were conditioned by FOM or BO. **(B)** The analysis of FOM and BO effect on individual cell subpopulations: endothelial cells, astrocytes, hOPCs, and OLs. The graph presented the means ± SD from three independent experiments. The differences between BO and FOM supplementation *vs*. adequate group in medium were calculated using the paired t-test.

At the beginning, we set the growth factor baseline on their content in the medium of the BBB construct with OLs (**Figure 3A**). We noted that the BBB construct is characterized by all tested factors, whose sources are astrocytes and ECs for IGF-1, astrocytes, hOPCs, and OLs for FGF-2, and hOPCs and OLs for BDNF (**Figure 3B**). Although we observed a detectable concentration of CNTF in the BBB construct with OLs, we did not observe it in the analysis of separated cell populations, pointing to the necessary cell cross-talk that initiates the secretion of this particular factor (**Figures 3A, B** low panel).

Next, we examined the result of FOM and BO supplementation on the concentration of growth factors released during polarization of hOPCs to mature OLs. The FOM supplementation of BBB, opposite to BO, resulted in increased concentration of IGF-1 (550 pg/ml in medium *vs*. 1,150 pg/ml in FOM supplementation), BDNF (0.69 pg/ml in medium *vs*. 1.85 pg/ml), and CNTF (90 pg/ml in medium *vs*. 130 pg/ml). Contrary to the growth factors mentioned above, we noted decreased concentration of FGF-2 during supplementation of BO and FOM (83 pg/ml in medium *vs*. 63 pg/ml in FOM supplementation and 69 pg/ml in BO supplementation) (**Figure 3A**). Taking into consideration ECs, astrocytes, OPCs, and OLs, the analysis of direct influence of FOM and BO on individual cell subpopulations revealed a significant increase of IGF-1 only in mature OLs exposed to FOM (21 pg/ml for medium *vs*. 51 pg/ml). In a similar comparison, we noted decreased concentration of FGF-2 in astrocytes (42 pg/ml medium *vs*. 32 pg/ml for BO and 21 pg/ml for FOM), in hOPCs (78 pg/ml medium *vs*. 52 pg/ml for BO and 69 pg/ml for FOM), and in OL culture medium (85 pg/ml medium *vs*. 52 pg/ml for BO and 67 pg/ml for FOM) after exposition to both tested oils. In turn, the analysis of BDNF concentration in the supernatants points to hOPCs and OLs, as these cells respond with increased synthesis after exposition to FOM and BO. We observed the following: 1.3 pg/ml in medium *vs*. 2.05 pg/ml for FOM and 2.4 pg/ml for BO in OPC culture supernatants and 1.05 pg/ml in medium *vs*. 1.4 pg/ml for FOM and 1.35 pg/ml for BO in OL culture. Additionally, we noted undetectable CNTF concentration in all investigated cell populations (**Figure 3B**).

### The comparison of free fatty acid receptor expression revealed the highest expression of FFAR4 on the oligodendrocyte cell line

**Figure 4:**
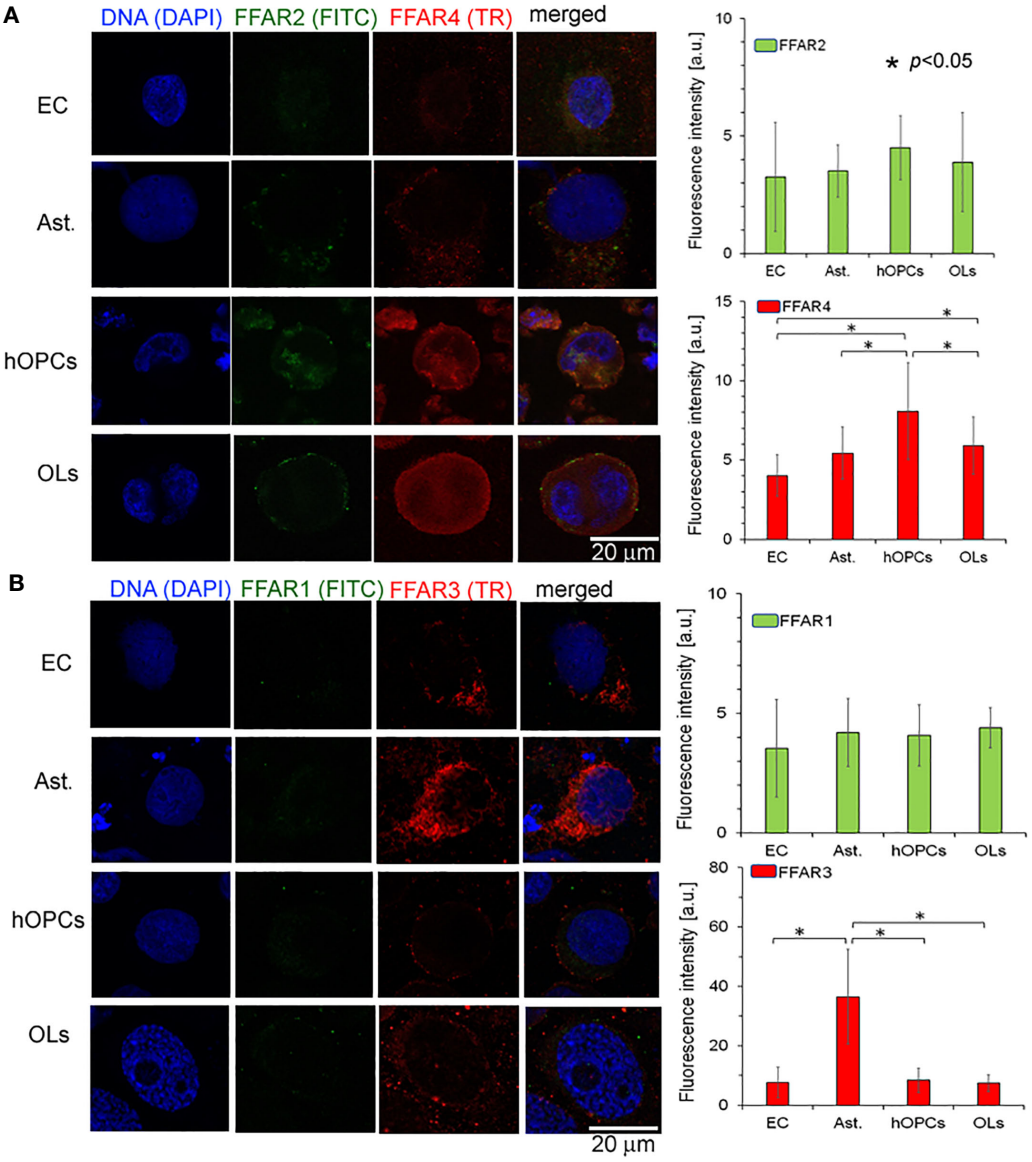
OPCs and mature OLs, contrary to BBB cells, are characterized by a high expression of FFAR4, receptors for long-chain saturated and unsaturated FA. In turn, a high expression of FFAR3, receptors recognized for short-chain fatty acids, is observed on astrocytes (Ast.). **(A)** The ICC analysis of FFAR2 (green pseudocolor) and FFAR4 (red) expression on separated cell cultures of ECs, Ast., hOPCs, and mature OLs. **(B)** ICC double-labeling of FFAR1 (green pseudocolor) and FFAR3 (red) on ECs, Ast., OPCs, and OLs. The bars represent average fluorescence intensity ± SD calculated from three independent experiments using at least 100 single cells for each test. The differences between different cell populations were calculated using the one-way ANOVA test and Scheffe’s test.

In the next set of experiments, we analyzed all known FFAR expressions on individual cell lines: astrocytes, ECs, hOPCs, and OLs. Using the ICC method, we revealed a higher expression of FFAR4 on hOPCs and OLs in comparison to both ECs and astrocytes. We noted that hOPCs are characterized by approximately 95% higher expression of FFAR4 compared to ECs and 56% compared to astrocytes. We noted FFAR4 fluorescent intensity of 4.1 a.u. in ECs, 5.1 a.u. in astrocytes, and 8.0 a.u. in hOPCs. Moreover, we observed a statistically significant decrease at about 28% of FFAR4 expression on OLs compared to hOPCs that could suggest a receptor pathway for fatty acids during the hOPC maturation (**Figure 4A**). We did not observe any differences in FFAR1 expression, another receptor for long-chain fatty acids (LCFAs), in any of the tested cell lines (**Figure 4B**). The analysis of FFAR2 and FFAR3, activated by short-chain fatty acids, revealed a high expression of FFAR3 on astrocytes compared to ECs, hOPCs, and OLs (**Figures 4A, B**).

### The change in the lipid composition points to the activation of nervonic acid synthesis pathway during human oligodendrocyte precursor cell maturation

**Figure 5:**
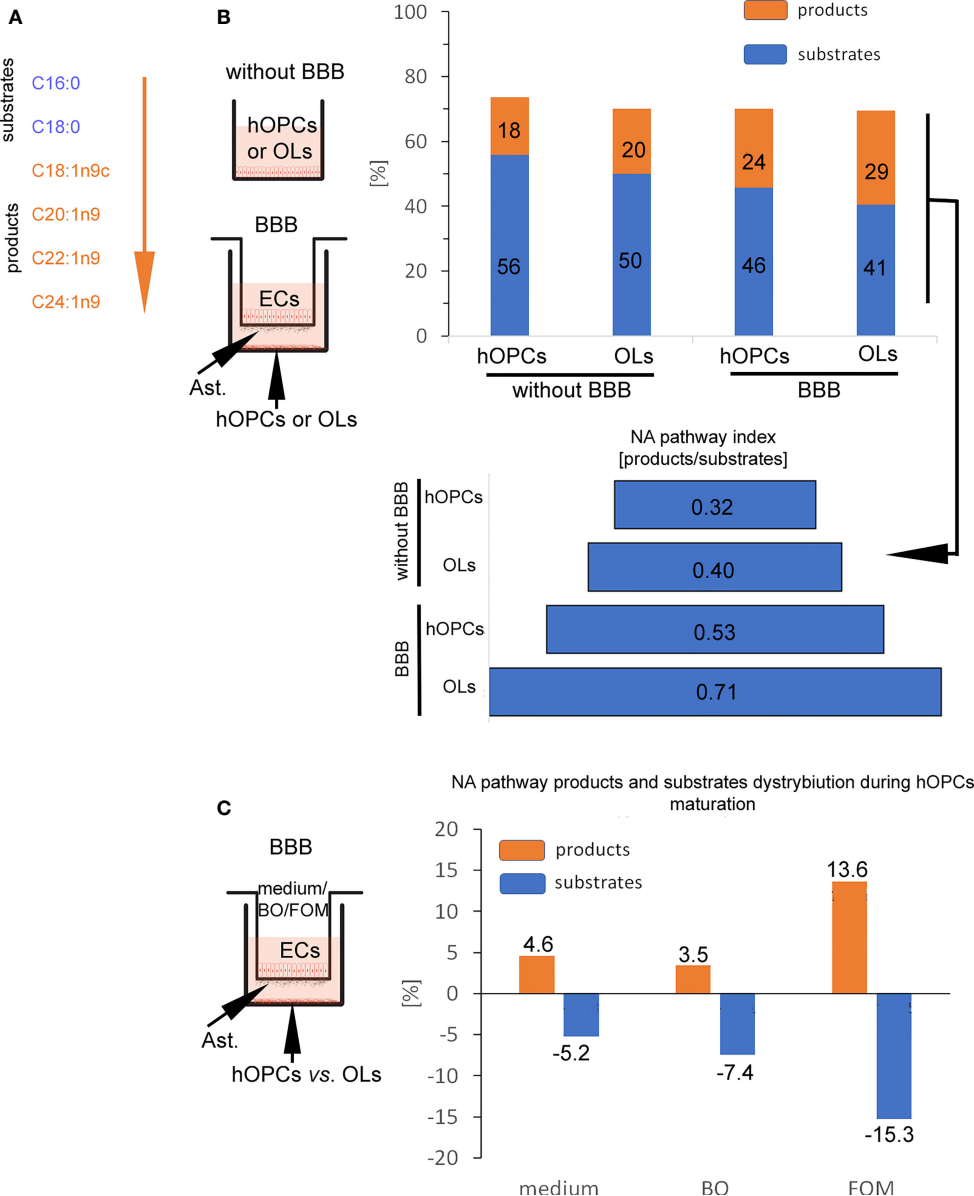
BBB cells supplemented with FOM enhanced NA synthesis by mature OLs. **(A)** The division into substrates: C16:0, C18:0 *vs*. products: C18:1n9c, C20:1n9; C22:1n9, C24:1n9 was set arbitrary based on the starting enzyme Δ9 desaturase, creating a carbon(C9)/carbon(C10) double bond. **(B)** BBB cells initiate lipid metabolism to NA synthesis. The distribution of substrates and products involved in NA synthesis by OPCs and OLs is presented as the percentage of all lipids and as NA pathway index. Data are presented as the mean calculated from three independent experiments. **(C)** The effect of BBB supplementation with FOM and BO on the NA pathway is presented as the percentage of decreasing substrates *vs*. product increment. Data are presented as the mean calculated from three independent experiments.

In the next step, GC/MS analysis was used to follow the changes in lipid composition during hOPC maturation. We checked the lipid profile of hOPCs and OLs in both systems, with and without BBB as well as individual populations of ECs and astrocytes exposed to FOM and BO. In this analysis, we focused on the influence of oils on the NA [C24:1(n-9)] pathway synthesis as the most important myelin lipid component. For better visualization of the NA synthesis process, we arbitrarily divided all of the NA pathway factors into substrates and products (**Figure 5A**). Chain elongation from C16:0/C18:0 substrates to C20:1(n-9)/C22:1(n-9)/C24:1(n-9) products has been set as the division point. The first important observation is that BBB cells influence the lipid profile in OLs, initiating NA synthesis. We revealed the increase in the percentage of products from 20% without BBB to 29% with BBB construct. In particular, we noted 0.32 NA pathway index (products/substrates) that characterized hOPCs without BBB *vs*. 0.53 with BBB construct. In mature OLs, we noted 0.40 NA pathway index without BBB *vs*. 0.71 with BBB (**Figure 5B**). A higher NA pathway index is the result of the appearance of more products with a simultaneous reduction in substrates (**Supplementary Table S1**). This experiment emphasizes the key role of EC/astrocyte forming the BBB in the remyelination process. Additionally, the analysis of NA substrate/product distribution during hOPC maturation revealed that only FOM supplementation increased the percentage of products by about 9.1% compared to that of control and by about 10.2% compared to that of BO (**Figure 5C**). The increase of products corresponds to the decreasing percentage of substrates by about 10.0% compared to that of control and 7.8% compared to that of BO (**Figure 5C**).

Considering the crucial role of BBB in the improvement of remyelination, we also checked changes in the lipid profile of individual BBB cells (**Supplementary Table S1**). After direct incubation of astrocytes and ECs with FOM, we can observe the increase of NA lipid products [oleic acid C18:1(n-9); cis-11-eicosenoic acid C20:1(n-9); erucic acid C22:1(n-9); nervonic acid C20:1(n-9)] in these cells compared to their undetectable level in the control group. Simultaneously, we noted the decrease of substrates such as palmitic acid (C16:0) and stearic acid (C18:0) during NA synthesis.

Moreover, since we do not observe squalene (present only in fish oils) and increased amount of DHA/EPA in the lipid profile of hOPCs and OLs with the use of the BBB, we can conclude that oils with LCFAs cannot diffuse through the membrane, and the effects on myelin protein synthesis and the NA pathway are indirectly related to stimulation *via* FFARs.

### Fish oil mixture and borage oil supplementation affects blood–brain barrier tight junction proteins maintaining the integrity of endothelial cells

In this experiment, we checked whether FOM and BO, as active components regulating EC activity, influence the BBB integrity. TJs that are under the control of adhesive molecules, such as CLDN-5 and VEC, form complexes in the space between ECs of the BBB, creating a highly regulated microenvironment for neural homeostasis.

**Figure 6:**
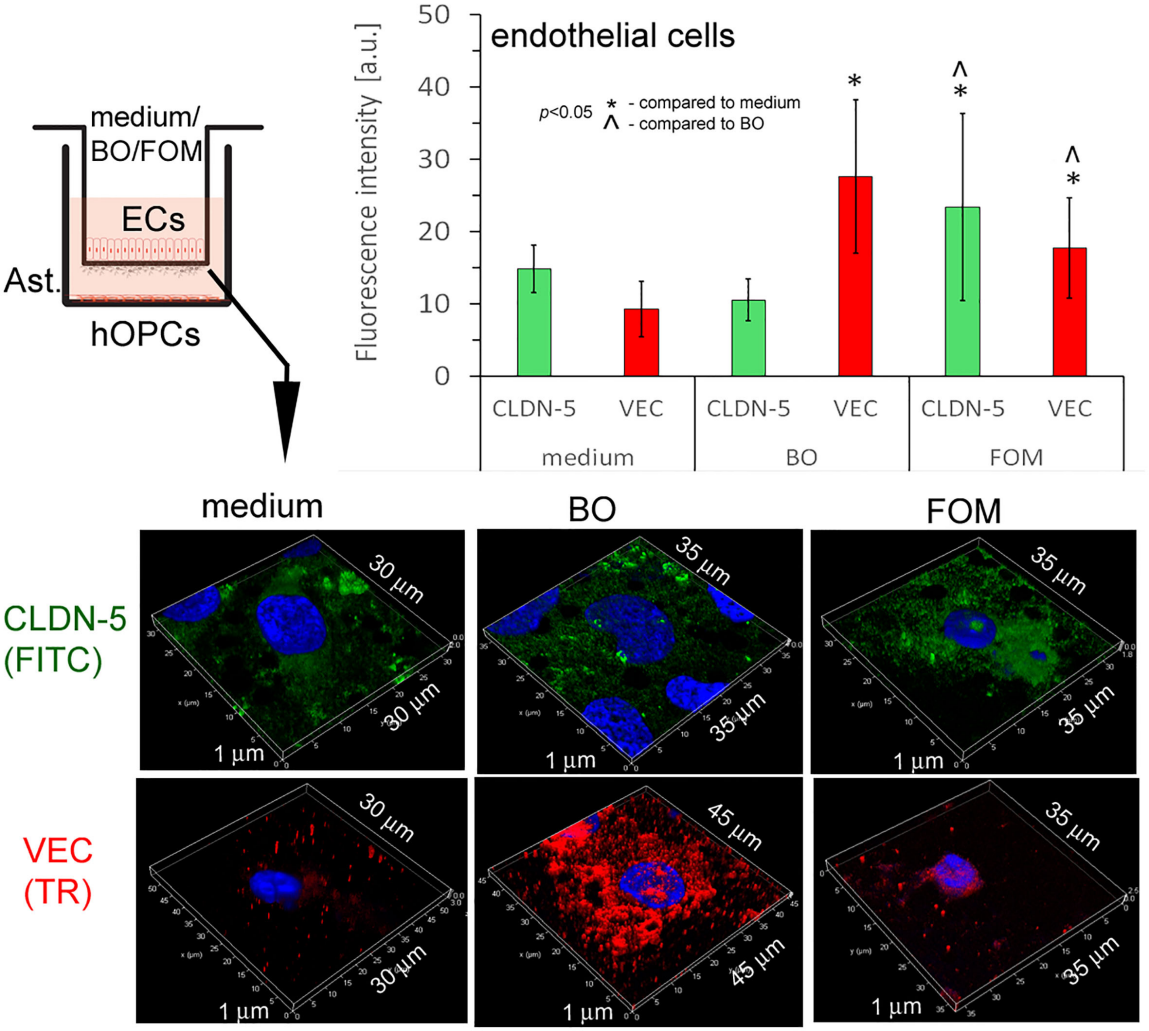
BBB cells supplemented with FOM increased claudin-5 (CLDN-5) and VE-cadherin (VEC) expression on endothelial cells (ECs), enhancing tight junction. ICC double-labeling for CLDN-5 (green pseudocolor) and VEC (red) on ECs with/without exposition to FOM and BO. ICC staining was performed after the membrane was cut from the Transwell inserts. The bars represent average fluorescence intensity ± SD calculated from three independent experiments using at least 100 single cells for each test. The differences between differently conditioned cells were calculated using the one-way ANOVA test and Scheffe’s test.

In this experiment, we used cut membranes from the inserts forming the BBB for double-labeling of CLDN-5 and VEC. Using the ICC method, we demonstrated that FOM supplementation affected the expression of both investigated molecules in ECs compared to that of control. We observed a significant increase in fluorescence intensity in CLDN-5 expression from 15 a.u. (medium) to 24 a.u. (FOM supplementation) and in VEC expression from 10 a.u. to 19 a.u., respectively. Moreover, BO also influenced VEC, increasing its expression to 28 a.u. compared to 10 a.u. for control, but opposite to FOM, BO did not affect CLDN-5 expression (**Figure 6**). Therefore, we postulated that FOM possesses a much positive effect on BBB integrity compared to BO.

### The comparative analysis of lipids in fish oil mixture *vs*. borage oil by RP-LC-Q-Orbitrap HRMS

**Figure 7:**
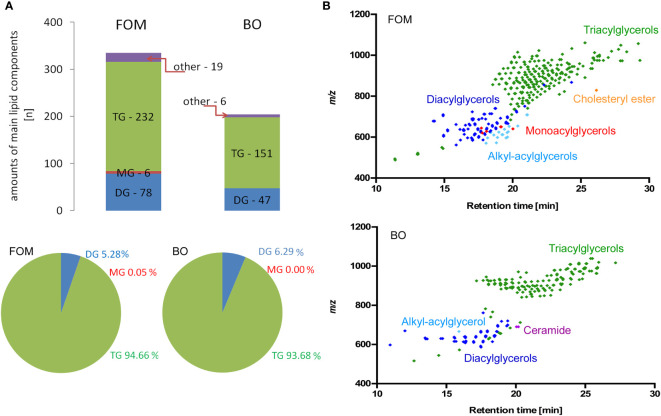
The comparative analysis of lipid species in FOM *vs*. BO by RP-LC-Q-Orbitrap HRMS technique displayed significantly more compounds in FOM compared to BO, despite similar percentages of all TG, DG, and MG in both oils. **(A)** The number and percentage of the lipid species divided into main lipid categories: MG, monoacylglycerol; DG, diacylglycerol; TG, triacylglycerol. **(B)** Retention time (RT) *vs*. mass-to-charge ratio (*m/z*) plots for lipid species identified in FOM and BO using RP-LC-Q-Orbitrap HRMS.

The last question we addressed concerns the differences in the lipid species composition between fish oil and borage oil. Although we have chosen two oils with a similar percentage of compounds involved in NA synthesis and supposed to exert a similar effect on BBB cells and OLs, the positive effect on NA and myelin peptide synthesis has been observed only for FOM supplementation. This observation suggests that there must be other accompanying compounds that can modulate cell metabolism. Therefore, we applied RP-LC-Q-Orbitrap HRMS technique that enables the analysis of lipid species without their derivatization to FA methyl esters, which is necessary for GC-based separation. For this reason, it was possible to analyze the FAs built into their primary esterified lipid structures. The data showed that FOM contains significantly more lipid species than BO (335 FOM *vs*. 204 BO). The spectacular differences in species number mainly concerned triacylglycerols (TGs) (232 FOM *vs*. 151 BO) and diacylglycerols (DGs) (78 FOM *vs*. 48 BO) (**Figure 7**; **Supplementary Table S2**), despite that there were no significant differences in the percentage of TG (94.7% FOM *vs*. 93.7% BO) and DG (5.3% FOM *vs*. 6.3% BO).

## Discussion

The insufficient process of remyelination, observed in different neurodegenerative disorders, is connected to forming the thinner structures of myelin sheaths (25). The properly functioning myelin sheath contains about 20%–30% of dry matter basis proteins and 70%–80% of lipids. The maintenance and unremitting synthesis of proper components for myelin sheaths including proteins (26) and lipids are tightly correlated. It seems probable that both the mutual proportions of proteins to lipids and the synthesis time of individual compounds are critical for sufficient remyelination. This process is orchestrated by the microenvironment mainly created by BBB cells delivering the necessary components for myelin synthesis and OPCs themselves. One of the important questions considers the mechanism of OPC myelin peptide synthesis *via* the BBB cells and their conditioning by natural oils that mimics the influence of a lipid diet on the CNS. Taking into account the high content of lipids in the myelin structure, we postulate two possibilities: stimulation of the BBB cells and OLs by FFAs *via* receptors or direct incorporation of FAs into cells. Recent studies have proven that FFARs are nutrient sensors expressed in various tissues and cells with the ability to regulate both energy metabolism and immune response. FFARs have received considerable interest in the last few years indicating the possibility of using them as novel therapeutic targets in the regulation of metabolism and immune responses (27).

In this study, we tested the thesis that the intake of necessary substrates for myelin synthesis may support the natural process of CNS regeneration. In the first step, we underline the critical role of the BBB in initiating the synthesis of myelin peptides MBP, MOG, and PLP as well as nervonic acid—NA—during maturation of hOPCs to mature OLs. Next, we revealed that this physiological synthesis of both myelin components was significantly enhanced after BBB supplementation with fish oil, but not BO. In our experimental model, we noted that OPCs exposed to the environment created by BBB cells increased the synthesis of MBP, MOG, and PLP about twice and simultaneously polarized lipid metabolism on the NA pathway synthesis. Conditioning BBB cells with fish oil added in the area of ECs, forming the BBB together with the astrocytes during OPC maturation additionally, enhanced myelin peptide and NA synthesis by a further 20%. It is worth emphasizing that the observed effect was simultaneous for MOG, MBP, and PLP peptides as well as NA synthesis, suggesting that LCFAs present in oils probably act as natural components indispensable for the proper synthesis of myelin. Another important observation after FOM supplementation is the significantly greater percentage increase in the level of MOG, MBP, and PLP peptides than mRNA copies compared to the experiment without FOM. We observed the increase in MOG, MBP, and PLP peptides to be approximately 18%–26% as estimated by mRNA copies and 77%–120% as estimated by protein levels. The comparison of experiments with OPCs incubated with BBB cells *vs*. direct exposition without BBB and supplementation showed the opposite relationship: significantly greater increase in the percentage of mRNA (approximately by about 60%–160%) than in the protein level (approximately 16%–30%). In our opinion, reverse proportions are the result of the multistep synthesis of myelin. First, maturating OPCs are preactivated to synthesize myelin proteins by BBB cells that enhance MOG, MBP, and PLP transcription (results in increasing mRNA copies). In the next step, maturating OPCs and OLs must be stimulated by FAs to initiate translation of MOG, MBP, and PLP and simultaneously polarized to lipid metabolism leading to the enhancement of NA synthesis. This theory suggested that LCFAs present in natural oils may act as triggering factors. Therefore, in the next step of our research, we analyzed the expression of all known FFARs on BBB cells, maturating OPCs, and mature OLs. Although the tested receptors covered all spectra of FFARs from those for short- to long-chain lipids and are present in all cell lines tested, we focused on FFAR1 and FFAR4 as receptors for LCFAs (characterized by 12 or more carbon atoms). We noted a high expression of FFAR4 on OPCs and mature OLs in comparison to ECs or astrocytes. Despite that FFAR4 expression has been detected within the CNS, its role remains poorly understood. In human and mice, FFAR4 ligation by LCFAs results in an intracellular increase of Ca^2+^ level and activation of signal-regulated kinases 1 and 2 (ERK1/2) (28). Several studies show that ERK1/2 activation in OPCs is critical for proper myelination during CNS development (29–31), and maintaining their activation is necessary for proper remyelination in adult animals (30, 32). In this context, high FFAR4 expression on hOPCs points to their privileged role in coordinating myelin synthesis as triggering factors, recognizing the presence of free LCFAs in the extracellular space, essential for myelin synthesis. We also noted that OPCs and OLs possess FFAR1 activated by ligation medium (6–12 carbon atoms) and long-chain free acids, but opposite to FFAR4, they have greater affinity to palmitoleic acid [C16:1(n-7)], α-linoleic acid [C18:3(n-3)], and DHA [22:6(n−3)] (33). Ma et al. (34), using immunohistochemical and Western blot analysis, have shown that FFAR1 is widely expressed in neuronal cells within the cerebral cortex, hippocampus, amygdala, hypothalamus, cerebellum, and spinal cord. They also reported that DHA induced neuronal differentiation and enhanced neuronal growth and branching in cultured rat neuronal stem cells transfected with FFAR1 genes (35). FFAR1 activated by DHA induces phosphorylation of cAMP response element-binding protein (CREB) transcription factor, which regulates the expression of genes promoting synaptic and neuronal plasticity, including BDNF genes (36). Although these results were obtained in human neuroblastoma cells, in our experiments, we noted the expression of FFAR1 in all of the cell populations tested and a high concentration of BDNF in FOM-conditioned hOPC/OL culture supernatants that contain 20% of DHA but not after supplementation with BO without DHA. BDNF gives a signal for initiating the remyelination process with the engagement of the cross-talk between neurons and myelin-producing OLs. OL-derived BDNF is responsible, *inter alia*, for neuronal survival, growth in CNS, and synaptic transmission (37). Based on the literature and our obtained data, we postulated that activation of FFAR1 and FFAR4 by long free fatty acids is essential to initiate the synthesis of proteins and lipids forming the myelin sheath.

Other relevant factors possessing neurodegenerative effects are IGF-1 and CNTF. In our research, we noted that BBB supplementation with FOM, but not BO, results in a high concentration of IGF-1 and CNTF in culture supernatants. One of the most important is IGF-1 with a potent trophic effect in the motor and sensory neurons and on neuronal development and regeneration (38–42). IGF-1 and CNTF promote neurite outgrowth and induce neuronal survival and differentiation (43, 44). Since the analysis of individual subpopulations conditioned by FOM or BO did not point to a specific subpopulation as a main source of IGF-1 and CNTF, we concluded that other unknown mechanisms must be involved in the increased synthesis of these growth factors during cooperation of BBB cells with maturating OPCs. Contrary to promyelinating growth factors, FGF-2 inhibits myelination by OLs (45, 46). We noted that BBB supplementation by FOM and BO results in a decreased concentration of FGF-2 in culture supernatants (approximately 10% in BO supplementation and 15% in FOM). This effect was probably the result of decreased synthesis of FGF-2 by astrocytes, hOPCs, and OLs, as direct exposition on individual cell populations revealed spectacular FGF-2 decrease in culture supernatants of these cell populations. We speculate that the decrease of FGF-2 observed in FOM and BO supplementation is a desired effect in the aspect of supporting the natural remyelination process.

The fluctuations within growth factors synthesized by BBB cells and OL line influence the BBB integrity. Recent studies reported that IGF-1 reduced the BBB permeability and decreased infarct volume in ischemia/perfusion rats (47). In turn, FGF-2 effectively protected the BBB from damage caused by intra-abdominal hypertension and traumatic brain injury (48). As the dysfunction of the BBB is thought to be a major hallmark of CNS disorders such as multiple sclerosis (49), an important aspect is to consider the influence of both tested oils on the BBB tightness excluding potential damage. The decrease in TJ proteins, CLDN-5, OCLN, and ZO-1, was observed in ischemic stroke and traumatic brain injury animal models (50, 51) and in TJ-related proteins in animal models of CNS inflammation such as multiple sclerosis (52, 53). We revealed that FOM supplementation “sealed” the BBB, positively affecting the expression of both CLDN-5 and VEC on ECs compared to control. In addition, BO increased VEC expression *vs*. control but not CLDN-5. Although we suggest an underappreciated spectrum of oil supplementation affecting OLs and the BBB, further studies with neutralization of particular growth factors and FFARs will bring us closer to understand the mechanism of naturally derived oil engagement in remyelination. In our studies, we used the BBB model without the pericyte cell line, which plays an important role in transcytosis and CNS immune infiltration (54, 55); therefore, we cannot exclude oil diffusion through the BBB and their direct influence on maturating OPCs as other mechanism.

The last question we addressed concerns different effects observed between the tested oils. Despite the similar composition of both oils in terms of main compounds, FOM showed a multidirectional potential affecting myelin protein synthesis, NA pathway, and growth factors compared to BO. The RP-LC-Q-Orbitrap HRMS technique revealed the significant differences in the number of lipid species between FOM and BO. We noted significantly more free fatty acids in fish oil *vs*. plant oil, especially in TG and DG. Another important property of naturally derived fish oil, contrary to BO, is the content of DHA in its composition, which possesses strong affinity to FFAR1. These FOM features can result in greater diversity in the response of FFAR1 and FFAR4 to more potential agonists involved in a spectrum of physiological processes.

Taken together, FOM supplementation with BBB engagement, in contrast to BO, tends to enhance many different factors involved in CNS regeneration. It can be considered as supportive therapy to stimulate resident precursor cell populations to repair demyelinated lesions next to a strategy based on cell replacement by transplanting OPCs. Although many clinical studies emphasize the beneficial effect of oil supplementation *via* their anti-inflammatory properties (56), our investigations performed on the BBB *in vitro* model present new features of fish oil with its positive effect on critical components for myelin synthesis, so important in the remyelination process.

## Data availability statement

The original contributions presented in the study are included in the article/**Supplementary Material**. Further inquiries can be directed to the corresponding author/s.

## Author contribution

PP designed BBB model, performed and analysed experiments (cytokine/chemokine/growth factors profiling assays in both groups, RNA isolation, mRNA concentration analysis, ELISA). NL prepared the manuscript (review and editing). SM and MW performed ICC analysis. RB performed gas chromatography-mass spectrometry analysis. KP performed RP-LC-Q-Orbitrap HRMS analysis. PL supervised and designed the study and experiments, interpreted the data and wrote the manuscript (original draft). MN mRNA concentration analysis, the manuscript writing and editing. All authors revised the manuscript. All authors contributed to the article and approved the submitted version.

## Funding

This work was supported by funds obtained from the cooperation between Medical University with The Marinex International Company (contract No. CRU: 0121-CSTT-2020).

## Conflict of interest

The authors declare a conflict of interest. This work was supported by funds obtained from the cooperation between Medical University of Lodz with The Marinex International Company (contract No. CRU: 0121-CSTT-2020). The Marinex International Company had no role in the design, execution, interpretation, and writing the manuscript.

## Publisher’s note

All claims expressed in this article are solely those of the authors and do not necessarily represent those of their affiliated organizations, or those of the publisher, the editors and the reviewers. Any product that may be evaluated in this article, or claim that may be made by its manufacturer, is not guaranteed or endorsed by the publisher.

## Supplementary material

Supplementary Figure 1 (A) The graph presents steps of preparing BBB model and OPC maturation. The verification of BBB model quality by ICC labelling for VE-cadherin (red pseudocolor) expression on endothelial cells and GFAP (green) – specific marker for astrocytes. (B) The analysis of OL purity during differentiation estimated by morphological shape in differential contrast microscopy (upper panel) and by ICC analysis. O4 - oligodendrocyte cell line marker, nestin - marker for immature OLs, MOG - mature cells, glial fibrillary acidic protein (GFAP) – specific marker for astrocytes were examined to check quality of oligodendrocytes used in experiments.

Supplementary Table 1 The effect of FOM and borage oil (BO) on the lipid composition in hOPCs performed by gas chromatography-mass spectrometry. The blue color indicates the main lipids involved in NA synthesis as substrates, yellow as products. Data are presented as the percentage of lipids from three independent experiments.

Supplementary Table 2 The lipid species identified in FOM and BO using RP-LC-Q-Orbitrap HRMS analysis. Data are presented as the percentage of lipids from two independent samples.
