# Supplementary figures and images for: Natural fish oil improves the differentiation and maturation of oligodendrocyte precursor cells to oligodendrocytes in vitro after interaction with the blood–brain barrier
Source: Front Immunol. 2022 Jul 22;13:932383. doi: 10.3389/fimmu.2022.932383 (PMC9353075; doi:10.3389/fimmu.2022.932383)

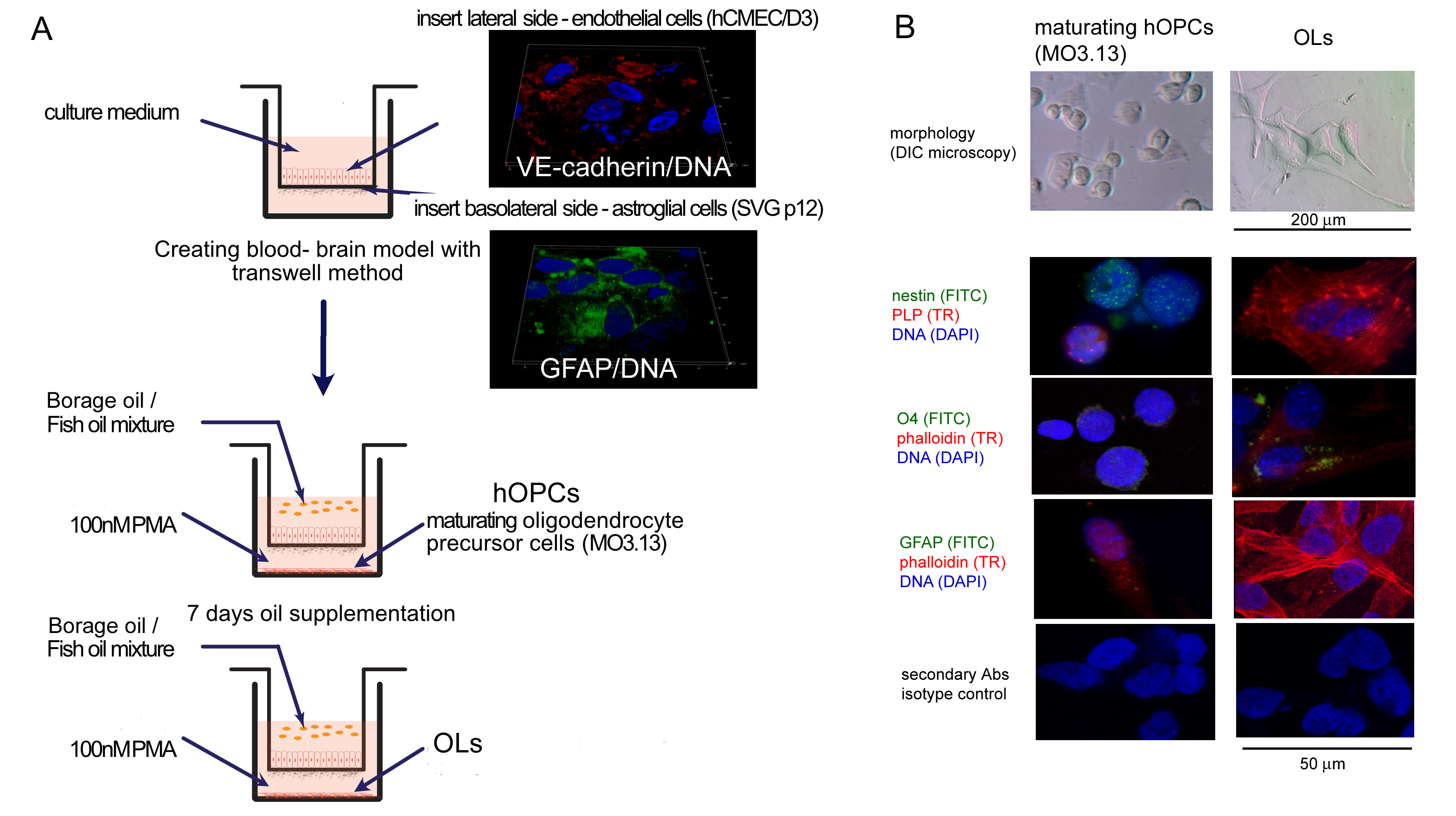

Supplement: Supplementary Figure 1 — (A) The graph presents steps of preparing BBB model and OPC maturation. The verification of BBB model quality by ICC labelling for VE-cadherin (red pseudocolor) expression on endothelial cells and GFAP (green) – specific marker for astrocytes. (B) The analysis of OL purity during differentiation estimated by morphological shape in differential contrast microscopy (upper panel) and by ICC analysis. O4 - oligodendrocyte cell line marker, nestin - marker for immature OLs, MOG - mature cells, glial fibrillary acidic protein (GFAP) – specific marker for astrocytes were examined to check quality of oligodendrocytes used in experiments. [file Image_1.tif]
